# N^ε^-Lysine Acetylation of the Histone-Like Protein HBsu Regulates the Process of Sporulation and Affects the Resistance Properties of *Bacillus subtilis* Spores

**DOI:** 10.3389/fmicb.2021.782815

**Published:** 2022-01-17

**Authors:** Jackson Luu, Connor M. Mott, Olivia R. Schreiber, Holly M. Giovinco, Melanie Betchen, Valerie J. Carabetta

**Affiliations:** ^1^Department of Biomedical Sciences, Cooper Medical School of Rowan University, Camden, NJ, United States; ^2^Department of Internal Medicine, Cooper University Hospital, Camden, NJ, United States

**Keywords:** post-translational modification (PTM), acetyl, acetylation, KAT, KDAC, endospore, bacteria, SASP

## Abstract

*Bacillus subtilis* produces dormant, highly resistant endospores in response to extreme environmental stresses or starvation. These spores are capable of persisting in harsh environments for many years, even decades, without essential nutrients. Part of the reason that these spores can survive such extreme conditions is because their chromosomal DNA is well protected from environmental insults. The α/β-type small acid-soluble proteins (SASPs) coat the spore chromosome, which leads to condensation and protection from such insults. The histone-like protein HBsu has been implicated in the packaging of the spore chromosome and is believed to be important in modulating SASP-mediated alterations to the DNA, including supercoiling and stiffness. Previously, we demonstrated that HBsu is acetylated at seven lysine residues, and one physiological function of acetylation is to regulate chromosomal compaction. Here, we investigate if the process of sporulation or the resistance properties of mature spores are influenced by the acetylation state of HBsu. Using our collection of point mutations that mimic the acetylated and unacetylated forms of HBsu, we first determined if acetylation affects the process of sporulation, by determining the overall sporulation frequencies. We found that specific mutations led to decreases in sporulation frequency, suggesting that acetylation of HBsu at some sites, but not all, is required to regulate the process of sporulation. Next, we determined if the spores produced from the mutant strains were more susceptible to heat, ultraviolet (UV) radiation and formaldehyde exposure. We again found that altering acetylation at specific sites led to less resistance to these stresses, suggesting that proper HBsu acetylation is important for chromosomal packaging and protection in the mature spore. Interestingly, the specific acetylation patterns were different for the sporulation process and resistance properties of spores, which is consistent with the notion that a histone-like code exists in bacteria. We propose that specific acetylation patterns of HBsu are required to ensure proper chromosomal arrangement, packaging, and protection during the process of sporulation.

## Introduction

Bacterial endospores are one of the most resilient cell-types, surviving in harsh environments without nutrition for a prolonged time. Endospores are formed during the process of sporulation, which is a survival strategy when spore-forming bacteria encounter severe environmental stresses or starvation. In *Bacillus subtilis*, there are seven stages of sporulation, with each step controlled by specific regulatory cascades in a coordinated temporal pattern (Ryter et al., 1966; Piggot and Coote, 1976). Early steps in this process involve the replication of the chromosome, asymmetric septum formation and the coordinated packaging of the DNA into the smaller forespore compartment. In the later stages of sporulation, the developing spore is engulfed and the outer layers of the cortex and coat proteins are assembled. Finally, mother cell lysis occurs and the mature spore is released (Higgins and Dworkin, 2012; Cho and Chung, 2020).

Spores have little to no metabolism and are more resistant than vegetative cells to stresses such as heat, desiccation, radiation, and oxidizing agents (Mason and Setlow, 1986; Banks et al., 1988; Russell, 1990; Setlow and Setlow, 1995; Loshon et al., 1999; Setlow, 2006). Spores can accumulate damage to proteins and DNA during extended periods of dormancy (Popham and Bernhards, 2015), and small amounts of accrued DNA damage is repaired once the spore germinates (Setlow et al., 2003; Moir and Cooper, 2015). However, accumulation of excessive damage is overwhelming and leads to reduced survival during germination (Setlow, 1995; Setlow and Setlow, 1996). Thus, there are many mechanisms to protect important biomolecules from damage during dormancy. To protect the DNA, the cell produces the α/β-type small acid-soluble proteins (SASPs). The SASPs are abundant spore proteins that are synthesized in the forespore and coat the chromosome to protect the DNA from environmental insults, such as ultraviolet (UV) radiation (Setlow et al., 1982; Mason and Setlow, 1986). SASP binding to DNA induces positive supercoils and increases persistence length (Nicholson et al., 1990; Griffith et al., 1994). Additionally, SASPs bound to DNA induce changes in UV photochemistry, namely by forming a spore photoproduct when exposed to UV radiation rather than thymine dimers, which are bulky and destabilizing lesions (Setlow and Setlow, 1987; Nicholson et al., 1991).

Clearly, chromosomal compaction is necessary to physically package the DNA into the smaller forespore compartment. The bacterial chromosome is compacted and organized by the action of the nucleoid-associated proteins (Dillon and Dorman, 2010). The essential, histone-like protein HBsu is a member of the widely conserved HU-family of proteins, which are largely responsible for chromosomal compaction and coordination of DNA transactions (Micka and Marahiel, 1992; Klein and Marahiel, 2002). While lacking sequence or structural homology, the HU-family is generally considered to represent functional homologs of eukaryotic histones. HBsu is present in mature spores and is involved in the organization of the SASPs (Ross and Setlow, 2000). In developing spores, HBsu colocalizes with the SASPs in the nucleoid region and modulates SASP-mediated properties. These modulatory effects, such as amelioration of SASP-mediated increases in DNA persistence length and opposing negative DNA supercoiling, suggested that HBsu is a primary modulator of SASP-mediated DNA alterations (Ross and Setlow, 2000). Taken together, these data indicate that HBsu may be a critical regulator during the process of sporulation.

N^ε^-lysine acetylation is a ubiquitous, regulatory post-translational modification (PTM) that influences a variety of biological processes in bacteria (Carabetta and Cristea, 2017; Christensen et al., 2019a,b; VanDrisse and Escalante-Semerena, 2019). Acetylation of lysine residues may alter protein–DNA interactions, inhibit enzymatic function, or change subcellular localization (Gu and Roeder, 1997; Glozak et al., 2005; Ishfaq et al., 2012; Li et al., 2012). Recently, we characterized the *B. subtilis* acetylome and found that the histone-like protein HBsu is acetylated at seven sites *in vivo* (Carabetta et al., 2016). Using substitution mutations that mimic the unacetylated and acetylated forms of HBsu, we showed that the inability to acetylate key lysine residues resulted in a more compacted nucleoid. Additionally, we showed that acetylation reduces the DNA binding affinity of HBsu, providing a potential regulatory mechanism (Carabetta et al., 2019). Acetylation of HBsu occurs enzymatically, which is carried out by acetyltransferases (KATs). The GCN5-like *N*-acetyltransferases (GNATs) are prevalent in bacteria, and catalyze the transfer of an acetyl group from acetyl-CoA to a target primary amine, such as on a lysine residue sidechain. Following a screen of predicted GNAT domain-containing proteins for defects in nucleoid compaction, we found that two GNATs, YfmK, and YdgE acetylate HBsu (Carabetta et al., 2019). In *B. subtilis*, there are two known lysine deacetylases (KDACs): SrtN of the NAD^+^-dependent sirtuin family and AcuC of the NAD^+^-independent, Zn^2+^-dependent family (Gardner and Escalante-Semerena, 2009). However, we currently do not know the mechanism of deacetylation for HBsu.

HBsu has been proposed to modulate the properties of the SASPs to ensure proper packaging of the chromosome into the spore. As HBsu is acetylated, and these PTMs are important for its chromosomal compaction function, we reasoned that acetylation of HBsu may also regulate sporulation. To test this hypothesis, we utilized substitution mutations that mimic the acetylated (K → Q) and unacetylated (K → R) forms of HBsu, and determined the influence of acetylation on overall sporulation frequency, and spore resistance properties. We found that specific HBsu acetylation patterns are required for proper sporulation. This suggests that HBsu is important for chromosomal dynamics during the early stages of spore formation, and that acetylation at specific sites regulates this process. As the SASPs are essential to protect the DNA from environmental insults, we predicted that altering HBsu acetylation might lead to improper chromosomal packaging, and therefore, to increased susceptibility to heat, UV and chemical stresses. To test this idea, we isolated *B. subtilis* spores from wild-type and mutant strains, and determined survival to exposure of each agent. We found that specific mutations led to decreases in survival to each stress. By using opposite mutations, we were able to ascertain if each site was more likely to be acetylated to some extent or unacetylated in the mature spore. Taken together, these data indicate that HBsu acetylation is required for proper protection of the spore chromosome, and this further supports the idea that HBsu and the SASPs work together to complete this task. Further work is required to fully elucidate the exact mechanism by which HBsu and the SASPs work together, and how acetylation modulates this process. We propose that specific acetylation patterns change the DNA-binding capacity or protein–protein interactions of HBsu, which is the underlying mechanism that regulates the process of sporulation.

## Materials and Methods

### Bacterial Strains, Media, and Growth Conditions

All *B. subtilis* strains are listed in Table 1. Deletion alleles of *sspA*, *sspB*, and *sspC* were acquired from the *Bacillus* Genetic Stock Center (BGSC). Each deletion was confirmed by sequencing performed by Eton Biosciences (Union, NJ, United States). To construct the *ydgE yfmK* double mutant, the erythromycin (ery) cassette, flanked by *loxP* sites, from the *ydgE* knockout strain was removed. The plasmid pDR244 (BGSC), which contains the Cre recombinase, was transformed into the strain by selection for spectinomycin, and screened for colonies that were ery sensitive. The plasmid pDR244 was then cured by growth at 45°C, as it contains a temperature sensitive origin of replication. The resulting strain was transformed with *yfmK*:ery DNA, as previously described (Albano et al., 1987). The strain was confirmed by PCR and sequencing. Deletion alleles of *srtN* and *acuC* were kindly provided by Jorge Escalante-Semerena. The *srtN*, *acuC*, and *ssp* alleles were transformed into the BD630 background (*his*, *leu*, *met*) as described (Albano et al., 1987). Liquid and agar Luria broth (LB), liquid minimal competence media, and Schaeffer’s sporulation media (DSM) were prepared as described previously (Schaeffer et al., 1965; Albano et al., 1987). Histidine, leucine, and methionine (50 μg/ml) were added to the competence media. Bacteria were grown at 37°C with aeration, and growth was monitored in a Klett colorimeter, when necessary. Antibiotics were added as appropriate and used at the following concentrations: 5 μg/ml ery, 5 μg/ml kanamycin (kan), 5 μg/ml chloramphenicol (cam), and 100 μg/ml spectinomycin (spc).

**TABLE 1:** Strains used in this study.

*B. subtilis* strains	Relevant genotype^1^	Source/references
BD630	*his leu8 metB5*	Lab strain
BD6861	*acuC:spc*	This study
BD7375	*srtN:cam*	This study
BD7199	*trpC2* Δ*ydgE:ery*	Carabetta et al., 2019
BD7203	*trpC2* Δ*yfmK:ery*	Carabetta et al., 2019
BD7484	*hbsK80R*	Carabetta et al., 2019
BD7493	*hbsK86Q*	Carabetta et al., 2019
BD7506	*hbsK86R*	Carabetta et al., 2019
BD8119	*hbsK37Q*	Carabetta et al., 2019
BD8120	*hbsK37R*	Carabetta et al., 2019
BD8147	*hbsK41Q*	Carabetta et al., 2019
BD8148	*hbsK41R*	Carabetta et al., 2019
BD8190	*hbsK18R*	Carabetta et al., 2019
BD8219	*hbsK18Q*	Carabetta et al., 2019
BD8333	*hbsK75R*	Carabetta et al., 2019
BD8387	*hbsK3R*	Carabetta et al., 2019
BD8398	*hbsK75Q*	Carabetta et al., 2019
BD8576	*hbsK80Q*	Carabetta et al., 2019
BD8577	*hbsK3Q*	Carabetta et al., 2019
VCB4	*sspA:ery*	This study
VCB5	*sspB:ery*	This study
VCB6	*sspC:ery*	This study
VCB56	Δ*ydgE* Δ*yfmK:ery*	This study

### Sporulation Frequency Determination

Sporulation frequency determinations were carried out as described previously (Carabetta et al., 2013). The sporulation frequency was calculated as heat-resistant colony forming units (CFUs)/ml divided by total viable cells (CFUs/ml). Sporulation frequency determinations were made at least three independent times.

### Isolation of Spores by Exhaustion

Spores were isolated as described previously (Costa et al., 2004), with the following modifications. Single colonies of freshly struck *B. subtilis* strains were inoculated into 20 ml of freshly supplemented DSM. Cells were incubated for 48 h, and harvested by centrifugation. Pellets were washed three times with cold water, and spores were isolated with a 20–50% Histodenz (Sigma-Aldrich) step gradient. Isolated spores were washed three times with cold water, and stored at 4°C.

### Spore Heat-Resistance Assay

Heat resistance assays were carried out as described previously (Mason and Setlow, 1986). Briefly, isolated spores were serially diluted into PBS (10 mM potassium phosphate, pH 7.4, 0.15 M NaCl). Prior to heating, dilutions were plated on LB plates to determine initial spore counts (CFUs/ml). Spore dilutions were incubated for 10, 20, and 30 min at 85°C. At each time point, 100 μl of each spore dilution was removed and plated onto LB agar. Plates were incubated at 37°C for at least 16 h overnight and the following morning, CFUs counted. Percent survival was calculated as heat-resistant CFUs/ml divided by the initial spore counts. Each assay was carried out at least three independent times.

### Spore Ultraviolet-Resistance Assay

Spores were diluted 1:10 into 2 ml of PBS. Prior to UV treatment, serial dilutions were plated on LB plates to determine initial spore counts (CFUs/ml). The diluted spores were added to an empty petri dish, and the lid was replaced. Spore suspensions were exposed to UV light for 1, 3, and 5 min using a CL-1000 Ultraviolet CrossLinker (UVP). The dose rate was set to 100 on the machine, corresponding to 10,000 μJ/cm^2^. The lids were used to shield and absorb the majority of the radiation, as the minimum setting on the machine was well above the LD_90_ for *Bacillus* spores (Setlow, 2006). The protocol was optimized using wild-type and *sspA* mutant spores, to be in agreement with previous studies (Mason and Setlow, 1986). Following UV exposure, the spores were serially diluted 10-fold into PBS, plated onto LB agar plates, and incubated overnight at 37°C. The following morning, CFUs were counted and percent survival determined as UV-resistant CFUs/ml divided by the initial spore counts. Experiments were carried out at least three independent times.

### Spore Chemical-Resistance Assay

Spore resistance to formaldehyde treatment was carried out as previously described (Loshon et al., 1999), with minor modifications. Prior to treatment, serial dilutions of spores were plated onto LB plates to determine initial spore counts (CFU/ml). Bacterial spores were incubated at 30°C in the presence of 2.5% (v/v) formaldehyde for 10, 20, and 40 min. At each time point, an aliquot was removed and diluted 1:10 into a 400 mM glycine solution, to neutralize the formaldehyde. Then, the spores were incubated at room temperature for 20 min. After incubation, the spore suspensions were serially diluted into PBS, and plated on LB agar. Plates were incubated for at least 16 h at 37°C, and the following morning, CFUs were recorded. Percent survival was calculated as formaldehyde-resistant CFUs/ml divided by the initial spore counts. Each experiment was carried out at least three independent times.

### Statistical Analyses

All statistical analyses were performed using GraphPad Prism 9. Data was presented as calculated percentages, and results were compared statistically using one- and two-factor ANOVAs. For sporulation frequency determinations, one-factor ANOVAs with *post hoc* Dunnett’s test were performed to compare each mutant strain to the wild-type. For analysis of all resistance assays, two-factor ANOVAs with repeated measures were performed at each time point. *Post hoc* Dunnett square analysis was performed to compare each mutant to the wild-type spores at each time point. Differences with a *p*-value of ≤0.05 were considered statistically significant.

## Results

### Specific HBsu Acetylation Patterns Are Required for Proper Sporulation

**FIGURE 1:**
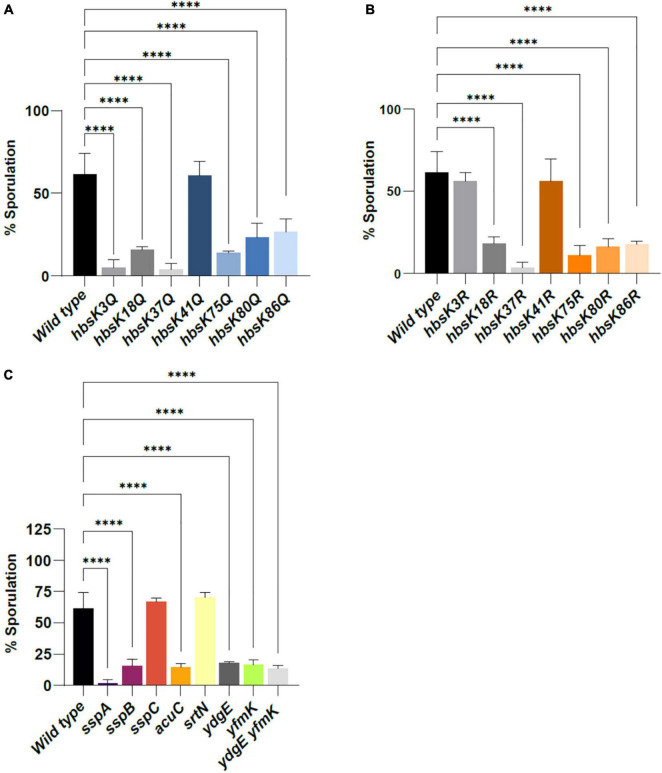
The effects of acetylation on sporulation frequency. *B. subtilis* strains were grown in sporulation media for 24 h, and subsequently exposed to heat for 30 min to kill vegetative cells. The sporulation frequency was calculated as heat-resistant colony forming units (CFUs)/ml divided by total viable counts (CFU/ml), pre-heat treatment. Bar graphs represent the average percentages determined from at least three independent replicates with error bars representing standard deviations. Strains used were as follows: **(A)** wild-type (BD630), *hbsK3Q* (BD8577), *hbsK18Q* (BD8219), *hbsK37Q* (BD8119), *hbsK41Q* (BD8147), *hbsK75Q* (BD8398), *hbsK80Q* (BD8576), *kbsK86Q* (BD7493). **(B)** *hbsK3R* (BD8387), *hbsK18R* (BD8190), *hbsK37R* (BD8120), *hbsK41R* (BD8148), *hbsK75R* (BD8333), *hbsK80R* (BD7484), and *hbsK86R* (BD7506). **(C)** *acuC* (BD6861), *srtN* (BD7375), *ydgE* (BD7199), *yfmK* (BD7203), *yfmK ydgE* (VCB56), *sspA* (VCB4), *sspB* (VCB5), and *sspC* (VCB6). Statistical analyses were performed using GraphPad Prism 9. One-factor ANOVAs and a *post hoc* Dunnett’s square analysis were used to determine statistical significance. A *p*-value of ≤0.05 was considered significant. ^****^p < 0.0001.

Sporulation is a regulatory process in *B. subtilis* to increase the probability of long-term survival. There are two likely steps during the sporulation process at which HBsu may be involved. During stage I of sporulation, the chromosome is replicated and then arranged in an *ori-ter-ori* alignment along the long axis of the cell, referred to as the axial filament (Webb et al., 1997; Wang et al., 2014). Then, in stage II, the asymmetric septum is formed, and initially about one third of the chromosome is located in the forespore compartment. Afterward, a translocase pumps the rest of the chromosome into the spore (Higgins and Dworkin, 2012; Cho and Chung, 2020). As HBsu is important for chromosome compaction and dynamics (Micka and Marahiel, 1992; Fernández et al., 1997; Köhler and Marahiel, 1997, 1998), it may be involved these early stages of sporulation. Previously, we showed that HBsu acetylation regulates chromosomal compaction (Carabetta et al., 2019). To investigate whether HBsu acetylation impacts the sporulation process, we utilized point mutations that mimic the acetylated (glutamine) and unacetylated (arginine) forms of HBsu and determined overall sporulation frequencies (Figure 1). *B. subtilis* strains were grown in sporulation media for 24 h, and exposed to heat for 30 min to kill vegetative cells. Sporulation frequencies were determined in triplicate as the number of CFUs following heat treatment divided by the initial viable counts. The viable counts for all strains were similar and are listed in Supplementary Table 1. The sporulation frequency for wild-type cells was 61.5 ± 12.6%. The sporulation frequencies of most of the acetyl mimic (Q) mutants were significantly reduced compared to wild-type, by 3- to 17-fold, with the exception of the *hbsK41Q* mutant (*p*-value = 0.9998, Figure 1A and Supplementary Table 2). The *hbsK37Q* mutant had a 17-fold reduction in sporulation frequency (*p*-value < 0.0001), and the most severe defect. We also determined sporulation frequencies of the unacetylated mimic (R) mutants. Five of the seven mutants (K18, K37, K75, K80, and K86) had significantly reduced frequencies compare to wild-type (Figure 1B and Supplementary Table 2), with the most severe defect again occurring with the *hbsK37R* strain (*p*-value < 0.0001). Since the “unacetylated” *hbsK3R* mutant was not significantly different from wild-type, this suggests that deacetylation of HBsu at K3 is required for the process of sporulation to occur properly. As the mimic mutants represent the extremes, with 100% locked in the acetylated or unacetylated state; our data suggests that some level of acetylation is required at K18, K37, K75, K80, and K86. Additionally, the acetylation state of K41 is not important for this process, as neither the Q nor R mutant showed any significant differences compared to wild-type.

Since it was previously proposed that HBsu modulates the properties of the SASPs (Ross and Setlow, 2000), we determined the sporulation frequencies of strains with deletions of the major SASP genes, *sspA* and *sspB*, and one minor SASP, *sspC*. The sporulation frequencies were significantly reduced when compared to wild-type in the *sspA* and *sspB* strains, with the most extreme phenotype observed for the *sspA* deletion (33-fold reduction, *p*-value < 0.0001, Figure 1C and Supplementary Table 2). The SASPs are synthesized and accumulate after the engulfment of the spore occurs (Tu et al., 2021), so they are not involved in the early sporulation process. The SASPs play a role in spore heat resistance [see below, (Setlow, 2006)], and heat treatment is used during the determination of sporulation frequency, which could partially explain the observed phenotypes. In addition, the heat treatment will kill spores that have not fully matured. Spores lacking both *sspA* and *sspB* have a delayed accumulation of dipicolinic acid (DPA), which is important for wet heat resistance, and this may also contribute the observed reduced sporulation frequency (Setlow et al., 2000). To further assess if acetylation was important for the sporulation process, we determined the sporulation frequencies of the two known HBsu acetyltransferases, YfmK and YdgE (Carabetta et al., 2019), and the known *B. subtilis* deacetylases, SrtN and AcuC (Gardner and Escalante-Semerena, 2009). In the *yfmK*, *ydgE*, and *ydgE yfmK* double mutants; there was a reduction in overall sporulation frequency, similar to that observed with some of *hbs* point mutants (*p*-value < 0.0001, Supplementary Table 2). This further suggested that specific acetylation patterns on HBsu are important for proper sporulation. The *acuC* mutant displayed a significant reduction (*p*-value < 0.0001, Figure 1C and Supplementary Table 2) in sporulation frequency, while the *srtN* mutant was not statistically different from wild-type. The specific deacetylase(s) for HBsu are currently unknown, and it is possible that the changes in sporulation frequency in the *acuC* mutant reflects changes in HBsu acetylation patterns. However, we cannot rule out that increases in acetylation on other unknown sporulation proteins are responsible for the observed changes.

### Acetylation of HBsu at Specific Sites Alters the Heat Resistance of Spores

**FIGURE 2:**
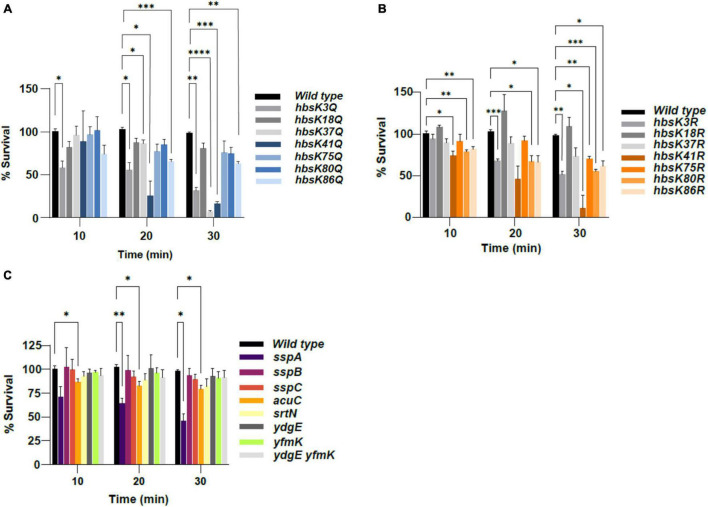
The effects of acetylation on heat resistance. *B. subtilis* spores were isolated following growth in sporulation media for 48 h. Spores were diluted and subsequently exposed to heat for a total of 30 min. At 10 min intervals, spores were plated for viable counts, and the survival percentage was calculated as heat-resistant colony forming units (CFUs)/ml divided by total viable counts (CFU/ml), pre-heat treatment. Bar graphs represent the average percentages determined from three independent replicates with error bars representing standard deviations. All time points were normalized to the zero time point for each strain (not displayed), which was set at 100%. The strains used in **(A–C)** were as described in the legend for Figure 1. Statistical analyses were performed using GraphPad Prism 9. Two-factor ANOVAs with repeated measures, and a *post hoc* Dunnett square analysis were used to determine statistical significance. A *p*-value of ≤0.05 was considered significant. *p < 0.05, ^**^p < 0.01, ^***^p < 0.001, ^****^p < 0.0001.

Spores display remarkable resistance or tolerance to destructive agents compared to growing cells (Setlow, 2006). When suspended in aqueous solutions, spores are more resistant to heat, called wet heat, than vegetative cells. The SASPs play a role in wet heat resistance, but many other factors such as core water content and mineralization are also important (Nicholson et al., 2000; Setlow, 2006). Since it was proposed that HBsu might modulate the properties of the SASPs during chromosomal packaging, we thought it possible that acetylation regulates the activity of HBsu during this process. Wild-type spores do not accumulate DNA damage in response to wet heat, but spores lacking the SASPs are killed by excessive DNA damage in the presence of wet heat, likely due to depurination (Setlow, 2006). Therefore, we expected mature spores from the *hbs* mutants to show increased sensitivity compared to wild-type spores when exposed to wet heat if their DNA was not properly packaged and protected. To begin, spores were isolated by exhaustion and purified using a Histodenz gradient, as described in section “Materials and Methods.” To assess whether acetylation alters heat resistance properties, spore suspensions were incubated with heat for 30 min, with survival determinations made every 10 min. The percentage of spore survival at each time point was calculated in triplicate. Wild-type spores were completely resistant to heat when exposed for 30 min (Figure 2 and see Supplementary Figure 1 for standard survival curves). For the *hbsK18Q*, *hbsK75Q*, and *hbsK80Q* mutants, there was no statistical difference in survival from wild-type spores (Figure 2A, Supplementary Figure 1A, and Supplementary Table 3). For the opposite R mutant spores, *hbsK80R* spores showed a small, but significant decrease in survival across the entire time course and *hbsK75R* showed a reduction in survival at the 30-min time point. The *hbsK18R* spores again displayed no significant difference from wild-type (Figure 2B, Supplementary Figure 1B, and Supplementary Table 3). These data suggest that a relatively high level of acetylation at K75 and K80 are needed for proper protection from heat, and that the acetylation status of K18 is not significant after the early stages of sporulation. The *hbsK3Q* mutant spores showed a significant decrease across the entire time course compared to wild-type, while the *hbsK3R* strain also showed marked decreases past 10 min (Figure 2, Supplementary Figure 1, and Supplementary Table 3). At K41 and K86, both Q and R mutant spores showed reduced survival to heat compared to wild-type spores, especially at the 30-min time points (Supplementary Table 3). This suggests that there is some intermediate level of acetylation of K3, K41, and K86 needed to confer long-term heat survival. Compared to wild-type spores, the *hbsK37Q* spores showed a significant decrease in survival at the later time points, with a large decrease in survival occurring after 30 min (<10%). Interestingly, the *hbsK37R* spores did not show significant differences from wild-type (Figure 2B, Supplementary Figure 1B, and Supplementary Table 2), suggesting that K37 is not acetylated under normal sporulation conditions. For the *ssp* deletion mutants, only *sspA* spores displayed significantly reduced survival from wild-type at 20 and 30 min (Figure 2C, Supplementary Figure 1C, and Supplementary Table 3). This is expected, since the SASPs are not the only determinant of wet heat resistance (Setlow, 2006). The defects in wet heat resistance seen among *hbs* mutants in general were larger than observed with the *ssp* mutants (Figure 2, Supplementary Figure 1, and Supplementary Table 3). This suggests that HBsu may play additional roles during sporulation, and may alter other important spore properties, such as core water content, DPA production or mineralization (Setlow, 2006), which influence wet heat resistance. Spores from *ydgE* and *yfmK* deletion strains and the double mutant were not reduced in survival, suggesting the possibility that these enzymes only influence HBsu during the sporulation process in the mother cell. *acuC* mutants displayed a significant, but modest reduction in survival, while *srtN* spores were similar to the wild type (Figure 2C, Supplementary Figure 1C, and Supplementary Table 3).

### Acetylation of HBsu Influences Spore Susceptibility to Formaldehyde

**FIGURE 3:**
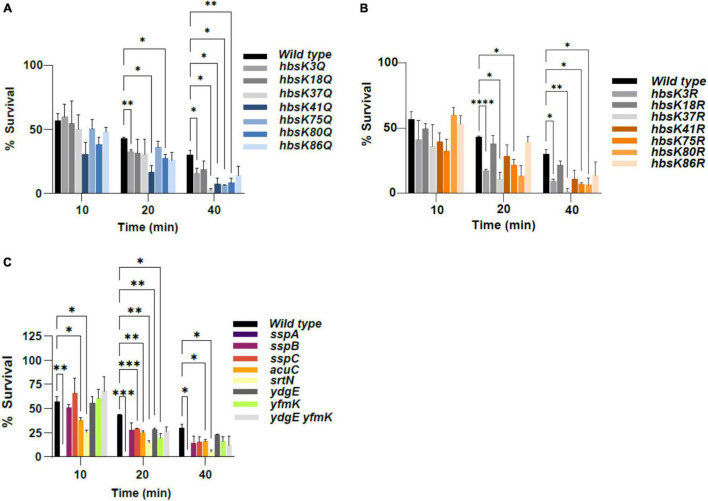
The effects of acetylation on formaldehyde resistance. *B. subtilis* spores were isolated following growth in sporulation media for 48 h. Spores were diluted and subsequently exposed to 2.5% formaldehyde for a total of 40 min. At 10, 20, and 40 min, spores were plated for viable counts, and the survival percentage was calculated as formaldehyde-resistant colony forming units (CFUs)/ml divided by total viable counts (CFU/ml), pre-treatment. Bar graphs represent the average percentages determined from at least three independent replicates with error bars representing standard deviations. All time points were normalized to the zero time point for each strain (not displayed), which was set at 100%. The strains used in panels **(A–C)** were as described in the legend for Figure 1. Statistical analyses were performed using GraphPad Prism 9. Two-factor ANOVAs with repeated measures, and a *post hoc* Dunnett square analysis were used to determine statistical significance. A *p*-value of ≤0.05 was considered significant. *p < 0.05, ^**^p < 0.01, ^***^p < 0.001, ^****^p < 0.0001.

It is known that spores of *B. subtilis* are resistant to decontamination procedures, such as formaldehyde treatment. Formaldehyde reacts with thiols and amines, and kills bacteria by alkylating amino acids and ring nitrogen atoms in purine bases (Favero and Bond, 2001). The main contributor to this chemical resistance in spores are the SASPs (Loshon et al., 1999). We found that specific acetylation patterns of HBsu were necessary for proper heat resistance, and we reasoned it likely that altering acetylation patterns would also influence formaldehyde resistance, and there would be a smaller percentage of spore survival. Isolated spores were exposed to 2.5% formaldehyde for 10, 20 and 40 min. The percentage of spore survival at each time point was calculated in triplicate, and survival data are displayed in Figure 3 and Supplementary Figure 2. About 40% of wild-type spores survive following exposure to this level of formaldehyde for 40 min. For all of the *hbs* mutants, there were no significant differences from wild-type during short-term exposure to formaldehyde at 10 min (Supplementary Table 4). The *hbsK18Q* and *hbsK86Q* spores had small reductions in survival, but none were not significantly different from wild-type spores during the entire 40-min exposure (Figure 3A, Supplementary Figure 2A, and Supplementary Table 4). The same was true for the opposite R mutants, suggesting that the acetylation state at these sites is not important (Figure 3B and Supplementary Figure 2B). Most of the *hbs* mutants showed significant differences from the wild-type spores as exposure time increased. The *hbsK3Q*, *hbsK41Q*, and *hbsK80Q* spores were significantly decreased in survival at the later time points (Figure 3A, Supplementary Figure 2A, and Supplementary Table 4). The same was true for the *hbsK3R* mutant, suggesting that the acetylation level of K3 may be intermediate (Figure 3B and Supplementary Table 4). For the *hbsK80Q* and *hbsK41Q* mutants, there was a significant reduction in survival at the two later time points, and the corresponding R mutants showed small differences that did not reach statistical significance at these time points (Figures 3A,B, Supplementary Figures 2A,B, and Supplementary Table 4). The *hbsK80R* spores did have a decrease with statistical significance at the 40 min time point (Figure 3B and Supplementary Table 4, *p*-value = 0.0157), which suggests that this site has a low level of acetylation in wild-type spores. The *hbsK41R* spores were not significantly different from wild-type, and thus K41 is most likely unacetylated (Supplementary Table 4). The *hbsK37R* and *hbsK75R* spores also were significantly decreased at the two later time points (Figure 3B, Supplementary Figure 2B, and Supplementary Table 4). In the corresponding Q mutants, there only were significant differences at the maximum exposure time of 40 min, which indicates that some level of acetylation is necessary, but it is possible that higher stoichiometries negatively affect long-term survival. As expected, the most severe phenotypes was observed with the *sspA* mutant. In the *sspA* mutant spores, there were no survivors after 10 min of exposure. Both the *srtN* and *acuC* mutant spores showed significant differences from wild-type over the entire time course (Figure 3C, Supplementary Figure 2C, and Supplementary Table 4). As mentioned previously, the exact reason for these decreases in survival cannot be ascertained, but it is possible that it is due increasing acetylation at specific sites on HBsu. The *ydgE* and *yfmK* mutants were only significantly different from wild-type at 20 min, and did show a small reduction in survival at 40 min (Figure 3C and Supplementary Figure 2C), while not significant [*p*-values 0.2322 and 0.0689 (Supplementary Table 4), respectively]. However, the *ydgE yfmK* double mutant was not significantly different from wild type across the entire time course.

#### Acetylation of HBsu Increases Susceptibility to Ultraviolet Radiation?

**FIGURE 4:**
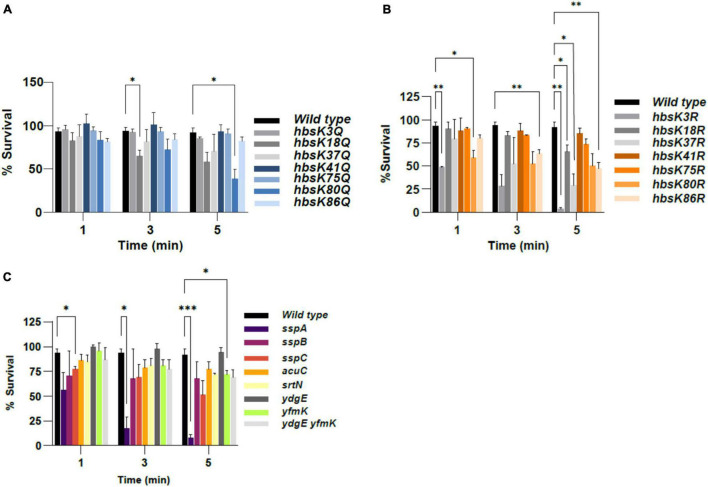
The effects of acetylation on UV resistance. *B. subtilis* spores were isolated following growth in sporulation media for 48 h. Spore suspensions were exposed to UV light for 0, 1, 3, or 5 min. The percent survival at each time point was calculated as UV-resistant colony forming units (CFUs)/ml divided by total viable counts (CFU/ml), pre-treatment. Bar graphs represent the average percentages determined from at least three independent replicates with error bars representing standard deviations. All time points were normalized to the zero time point for each strain (not displayed), which was set at 100%. The strains used in panels **(A–C)** were as described in the legend for Figure 1. Statistical analyses were performed using GraphPad Prism 9. Two-factor ANOVAs with repeated measures, and a *post hoc* Dunnett square analysis were used to determine statistical significance. A *p*-value of ≤0.05 was considered significant. *p < 0.05, ^**^p < 0.01, ^***^p < 0.001.

Spores are significantly more resistant than vegetative cells to irradiation with UV light. The SASPs are known to protect the DNA from damage caused by UV light, by changing the UV photochemistry (Setlow and Setlow, 1987; Nicholson et al., 1990, 1991; Griffith et al., 1994). To assess whether acetylation of HBsu affects UV susceptibility properties, spore dilutions were exposed to UV light for 1, 3, and 5 min. The percentage of spore survival at each time point was calculated in triplicate, and results are displayed in Figure 4 and Supplementary Figure 3. For the Q mutants, none displayed any differences from wild type after a 1-min exposure (Figure 4A, Supplementary Figure 3A, and Supplementary Table 5). The *hbsK18Q* spores showed a significant decrease in survival after 3 min and a reduction at 5 min, but not significant (*p*-value = 0.0652, Supplementary Table 5). The opposite *hbsK18R* spores were significantly different from wild type after 5 min of UV exposure, suggesting that the acetylation status at this site is likely not important (Figure 4B, Supplementary Figure 3B, and Supplementary Table 5). The *hbsK37Q* and *hbsK80Q* mutant spores survival was reduced across the entire time course, with a significant reduction at 5 min (Figure 4A, Supplementary Figure 3A, and Supplementary Table 5). As the *hbsK37Q* spores were similar to the wild type, this suggests that a relatively high acetylation stoichiometry is present at this site (Figure 4, Supplementary Figure 3, and Supplementary Table 5). The *hbsK80R* mutant also had a reduction in survival (Figure 4B and Supplementary Figure 3B), suggesting that an intermediate level of acetylation at this site exists in wild-type spores. The *hbsK86R* mutant was reduced at each time point, and was significantly different from wild type after 3 min of UV exposure, while the *hbsK86Q* mutant was not (Figure 4 and Supplementary Table 5), suggesting this site is normally acetylated, possibly at a high stoichiometry. As none of the K75 or K41 mutants differed significantly wild-type spores, the acetylation state at these two sites is not important. The *hbsK3Q* mutant was nearly identical to the wild type, but the opposite *hbsK3R* mutant was severely decreased across the entire time series, and at 5 min was reduced to a similar extent as an *sspA* mutant (Figures 4B,C). This suggests that K3 may be important for the regulation of the major SASP SspA. The *sspA* spores had the most severe phenotype, while *sspB* and *sspC* spores had mild decreases in survival that were mostly not significant (Figure 4C, Supplementary Figure 3C, and Supplementary Table 5). These observations are in agreement with previous work (Mason and Setlow, 1986). As seen with heat and formaldehyde resistance, there were no large differences observed in the *yfmK*, *ydgE*, or the double mutant (Figure 4C and Supplementary Figure 3C). The deacetylase mutants also did not display significant differences from wild-type spores.

## Discussion

HBsu is an abundant, nucleoid-associated protein that can be modified by acetylation (Carabetta et al., 2016). Previously, we demonstrated that one physiological function of acetylation of HBsu is to decondense the chromosome, and a likely mechanism by which this occurs is that acetylation reduces its DNA binding affinity (Carabetta et al., 2019). We have now demonstrated that acetylation of HBsu at specific sites is an additional regulatory mechanism of the sporulation process in *B. subtilis*. We found that K3 was likely not acetylated during this process and that some level of acetylation is needed at K18, K37, K75, K80, and K86 (Figure 1). The acetylation status of K41 was unimportant. The mostly likely stages of sporulation that could be affected by HBsu, given its known role in chromosome dynamics, are stages I and II. In stage I, the duplicated chromosome is stretched across the long axis of the cell, arranged in an *ori-ter-ori* orientation (Webb et al., 1997; Wang et al., 2014). As HBsu is one of the only nucleoid-associated proteins present in *B. subtilis* (Ohniwa et al., 2011), it is likely that it plays a role in axial filament formation, and could be responsible for arranging the chromosome in this manner. To test this idea, super resolution microscopy with fluorescently labeled chromosomal markers (Wang et al., 2014) and a *spoIIIE* deletion could be used. SpoIIIE is the translocase that moves the remainder of the spore chromosome across the asymmetric septum during stage II of sporulation, and deletion of this protein causes the cells to become blocked at axial filament formation (Wu and Errington, 1994, 1998). We would expect to see random organization of chromosomal loci in the *hbs* mutants, if HBsu acetylation was influencing this process. HBsu may also be important during or immediately following the chromosomal translocation process. SpoIIIE strips the DNA of RNA polymerase, transcription factors and chromosome remodeling proteins, like HBsu, during the translocation step (Marquis et al., 2008), so that the remaining two thirds of the DNA is naked when it enters the spore. However, the amount of HBsu present in the forespore is equivalent to that in the mother cell (Ross and Setlow, 2000). Therefore, HBsu must be reestablished at some point on the forespore DNA molecule, and perhaps acetylation regulates the timing or location of binding, which might influence SASP binding properties. Visualizing chromosomal structure in wild-type, *ssp* and *hbs* mutant spores by techniques such as Hi-C could address this possibility.

These observations raise further interesting questions. Currently, we do not know if different acetylated species of any protein exist in the cell. In other words, among the pool of HBsu in the cell, are there only singly acetylated species, or are there some species with two, three, or more modifications? Moreover, we do not have any information about the stoichiometry of acetylation at each individual site or the relative abundance of each species, if they exist. The stoichiometry at any particular site is likely dependent upon the growth phase and environmental conditions. We are currently developing a new mass spectrometry-based workflow to make such determinations on a single protein. As of now, multiple published mass spectrometry-based methods exist for quantification of the stoichiometry of the entire proteome (Baeza et al., 2014; Huang et al., 2015; Meyer et al., 2016; Weinert et al., 2017). It is of interest to determine how acetylation patterns and stoichiometry change during the process of sporulation. Another question worth exploring is how acetylation at specific sites regulates the sporulation process. It is likely that acetylation of lysine residues that directly contact the DNA will weaken the binding affinity. This could explain why deacetylation at K3, a site that likely contacts the DNA (Carabetta et al., 2019), is important. HU-family proteins are known to have a preference for structured DNA, such as bends and kinks (Pontiggia et al., 1993; Balandina et al., 2002). Perhaps SASP binding creates specific DNA structures that can only bind specific acetylated forms of HBsu. Another possibility is that these sites lie in protein–protein interaction interfaces. This may be especially true for those modification sites which likely do not contact the DNA, like K37 and possibly K75. Acetylation of a residue in a binding interface my cause a steric hindrance, and interfere with the interaction. The identities of such interacting proteins, if they exist, are not currently known, but a possibility could be the SASPs themselves. The SASPs increase persistence length (stiffness) of DNA, which is incompatible with the organization and compaction necessary to fit the DNA into the small spore compartment (Ross and Setlow, 2000). Perhaps HBsu–SASP complexes have different activities when bound together to DNA, and therefore acetylation might be a regulatory mechanism to control when these complexes form and therefore alter SASP properties. Finally, as HBsu is a histone-like protein, it is still possible that the acetylated forms of HBsu regulate gene expression in either the mother cell or the spore compartment. This possibility is suggested from the spore wet heat resistance experiments (Figure 2 and Supplementary Figure 1B), as multiple *hbs* mutants displayed larger defects than the *ssp* mutants. For example, alterations in the lipid composition of the highly impermeable spore inner membrane results in increased sensitivity to wet heat and oxidizing agents (Griffiths and Setlow, 2009; Leggett et al., 2012). If HBsu acetylation altered the expression of the lipid synthesis genes, such as *ugtP* [diacylglycerol (Jorasch et al., 1998)], *pssA* [phosphatidylserine (Okada et al., 1994)], or *ynwE* [cardiolipin (de Mendoza et al., 1993)], this could change the composition of lipids in the inner membrane, resulting in increased permeability and could explain the observed defects for both heat and formaldehyde resistance. Further work is required to address this possibility.

Once the mature spore is released, the DNA should be protected from extreme environmental insults. We wondered if the spores that were formed in these various mutants, even if the overall number was reduced, were normal. We thought it likely that the DNA was not properly packaged and condensed in the *hbs* mutants and we determined that this was true for wet heat, UV and formaldehyde resistance (Figures 2–4 and Supplementary Figures 1–3). As the short-term resistance properties of the mutant spores were altered, it will be interesting to assess their long-term survival capabilities, and specifically their ability to germinate. The most interesting observation of this data is that the acetylation patterns are different from those observed for the sporulation process. For example, K3 is likely unacetylated during the process of sporulation, and interestingly requires some level of acetylation for stress resistance (Figures 2–4 and Supplementary Figures 1–3). When these changes in acetylation patterns occur during this process is unclear, as they could happen during axial filament formation, during spore packaging, or at a later step. An interesting possibility is that there is expression of a KAT(s) or KDAC(s) that occurs exclusively in the spore compartment, perhaps under the control of a late sporulation sigma factor, like σ*^G^* (Fimlaid and Shen, 2015). This would allow for regulation of HBsu by acetylation to occur during different stages of the sporulation process. The *ydgE* and *yfmK* and double mutant spores were similar to wild-type, or had mild phenotypes in all conditions (Figures 2C, 3C, 4C). These mutant spores did show a significant reduction in sporulation frequency (Figure 1), suggesting that they might set the HBsu acetylation patterns early during the process, but are not the KATs responsible for changing the patterns later on. However, we previously identified three more uncharacterized KATs that might acetylate HBsu, YdhI, YokD, and YjbC (Carabetta et al., 2019). As little is currently known about the expression of these enzymes, it is possible that they are induced specifically in the spore compartment during the sporulation process. It is also possible that a deacetylase is induced in the spore compartment, which could also change the stoichiometry of acetylation. The two known *B. subtilis* deacetylases, SrtN and AcuC deacetylate AMP-intermediate forming enzymes, such as acetyl-CoA synthetase (Gardner and Escalante-Semerena, 2009). The exact mechanism of HBsu deacetylation remains unknown; however, in *Escherichia coli*, the sirtuin CobB deacetylates the HBsu orthologs HupA and HupB (AbouElfetouh et al., 2015). It is possible that SrtN is an HBsu deacetylase, but this must be confirmed. CobB has at least 50 substrates (AbouElfetouh et al., 2015), but the substrates of both SrtN and AcuC have not been determined. We found that *srtN* and *acuC* mutant spores showed significant decreases in formaldehyde survival, and *acuC* mutants also had decreased wet heat resistance (Figures 2, 3 and Supplementary Tables 3, 4). One possibility is that the decreases are due to changes in HBsu acetylation patterns. However, it is also possible that SrtN and AcuC have other substrates that are important for the sporulation process to proceed normally. These possibilities are not mutually exclusive. Further work is necessary to determine the substrates of SrtN and AcuC and understand the roles these enzymes play during the process of sporulation.

HBsu is part of the most widely conserved HU-family, and these proteins are generally considered to represent functional homologs of eukaryotic histones. Histone proteins contain long, unstructured N-terminal tails that are the sites of various PTMs, including lysine acetylation. The combination of these PTMs on histone tails is regulatory in nature, and constitutes the histone code (Bannister and Kouzarides, 2011; Rothbart and Strahl, 2014). These PTM combinations are hypothesized to regulate the interaction of the histone with DNA and other protein machines, and regulate most DNA processes, such as gene expression (Strahl and Allis, 2000). HBsu is acetylated at seven lysine residues, and we previously proposed that these modifications represent part of an analogous histone-like code in bacteria (Carabetta et al., 2019). Exploration of characterized acetylomes of multiple bacterial species revealed that this modification is evolutionarily conserved, even among distantly related bacteria (Carabetta, 2021). We previously determined that some level of acetylation at K3, K18, K41, K75, K80, and K86 was required for proper chromosomal compaction (Carabetta et al., 2019). If this truly represents a histone-like code, it is likely that most of the biological processes that HBsu influences would also be impacted by acetylation. We have now added sporulation to the list. It is possible that different acetylated species exist, and these influence which regions HBsu binds to on the chromosome. This could be an important regulatory mechanism to regulate SASP binding, and control the stiffness of the chromosome during spore packaging. In support of this idea, it was shown that both HBsu and the SASPs are present in the mature spore, and can bind simultaneously to the same DNA molecule *in vitro* (Ross and Setlow, 2000). It is possible that changing the ratios of acetylated species in the spore changes the HBsu binding locations on the chromosome, which could reduce the overall compaction or saturation with SASPs. This would explain our observed phenotypes with increased sensitivity to heat, UV and formaldehyde stresses. All together, our data lends further support that a bacterial version of a histone-like code exists.

## Data Availability Statement

The raw data supporting the conclusions of this article will be made available by the authors, without undue reservation.

## Author Contributions

JL, CM, OS, and HG performed all experiments, and analyzed and interpreted the data. MB performed the statistical analyses. MB and CM made the figures. VC designed the study, and analyzed and interpreted the data. All authors were involved in the writing and revisions of the manuscript.

## Conflict of Interest

The authors declare that the research was conducted in the absence of any commercial or financial relationships that could be construed as a potential conflict of interest.

## Publisher’s Note

All claims expressed in this article are solely those of the authors and do not necessarily represent those of their affiliated organizations, or those of the publisher, the editors and the reviewers. Any product that may be evaluated in this article, or claim that may be made by its manufacturer, is not guaranteed or endorsed by the publisher.
