# Supplementary material for: Nε-Lysine Acetylation of the Histone-Like Protein HBsu Regulates the Process of Sporulation and Affects the Resistance Properties of Bacillus subtilis Spores
Source: Front Microbiol. 2022 Jan 17;12:782815. doi: 10.3389/fmicb.2021.782815 (PMC8801598; doi:10.3389/fmicb.2021.782815)
Supplement: Supplementary file 1 [file Data_Sheet_1.PDF]

Supplementary Material

Contents

1 Supplementary Tables 2
1.1 Supplementary Table S1: Sporulation frequency viable count determinations......................2
1.2 Supplementary Table S2: Sporulation frequency p-values..3
1.3 Supplementary Table S3: Heat resistance p-values...4
1.4 Supplementary Table S4: Formaldehyde resistance p-values. 5
1.5 Supplementary Table S5: UV resistance p-values..6
2 Supplementary Figures..7
2.1 Supplementary Figure S1: Heat survival curves of spores from mutants that alter the acetylation state of HBsu...7
2.2 Supplementary Figure S2: Formaldehyde survival curves of spores from mutants that alter the acetylation state of HBsu. 8
2.3 Supplementary Figure S3: UV survival of spores from mutants that alter the acetylation state of HBsu. 9

1 Supplementary Tables

1.1 **Supplementary Table S1:** Sporulation frequency viable count determinations

	CFU/ml
**Wild type**	1.37E+08
hbsK3Q	1.44E+08
hbsK18Q	1.42E+08
hbsK37Q	2.19E+08
hbsK41Q	2.80E+08
hbsK75Q	2.47E+08
hbsK80Q	1.37E+08
hbsK86Q	9.87E+07
hbsK3R	2.15E+08
hbsK18R	1.87E+08
hbsK37R	1.81E+08
hbsK41R	2.77E+08
hbsK75R	2.61E+08
hbsK80R	2.09E+08
hbsK86R	1.71E+08
sspA	1.65E+08
sspB	1.73E+08
sspC	1.91E+08
yfmK	2.42E+08
ydgE	2.28E+08
ydgE yfmK	2.11E+08
acuC	1.34E+08
srtN	1.73E+08

Supplementary Table S1. A table of the corresponding viable counts following growth in sporulation media (DSM) for 24 hours. Displayed are the averages of three independent experiments.

1.2 Supplementary Table S2: Sporulation frequency p-values.

Sporulation Frequency	
Strain	p-value
hbsK3Q	<0.0001****
hbsK18Q	<0.0001****
hbsK37Q	<0.0001****
hbsK41Q	0.9998
hbsK75Q	<0.0001****
hbsK80Q	<0.0001****
hbsK86Q	<0.0001****

Sporulation Frequency	
Strain	p-value
hbsK3R	0.9237
hbsK18R	<0.0001****
hbsK37R	<0.0001****
hbsK41R	0.8680
hbsK75R	<0.0001****
hbsK80R	<0.0001****
hbsK86R	<0.0001****

Sporulation Frequency	
Strain	p value
sspA	<0.0001****
sspB	<0.0001****
sspC	0.8326
acuC	<0.0001****
srtN	0.2861
ydgE	<0.0001****
yfmK	<0.0001****
ydgE yfmK	<0.0001****

Supplementary Table S2. A table of the corresponding p-values for the data presented in Figure 1. *B. subtilis* strains were grown in sporulation media for 24 hours, and subsequently were exposed to heat for 30 minutes to kill vegetative cells. The sporulation frequency was calculated as heat-resistant colony forming units (CFU)/ml divided by total viable counts (CFU/ml), pre-heat treatment. Statistical analyses were performed using GraphPad Prism 9. One-factor ANOVAs and post-hoc Dunnett's tests were used to determine statistical significance**.** A p-value of ≤0.05 was considered significant. ****p<0.0001.

1.3 Supplementary Table S3: Heat resistance p-values.

Heat Resistance			
**Strain**	**Time (min)**		
	**10**	**20**	**30**
hbsK3Q	0.0152*	0.0221*	0.0019**
hbsK18Q	0.0796	0.0622	0.0931
hbsK37Q	0.9518	0.0307*	<0.0001****
hbsK41Q	0.9782	0.0414*	0.0003***
hbsK75Q	0.9597	0.0885	0.2604
hbsK80Q	0.9998	0.0763	0.0738
hbsK86Q	0.1197	0.0002***	0.0014**

Heat Resistance			
**Strain**	**Time (min)**		
	**10**	**20**	**30**
hbsK3R	0.5265	0.0003***	0.0039**
hbsK18R	0.0901	0.3886	0.5702
hbsK37R	0.1294	0.2338	0.1415
hbsK41R	0.0237*	0.0612	0.0286*
hbsK75R	0.5033	0.1697	0.0055**
hbsK80R	0.0051**	0.0174*	0.0004***
hbsK86R	0.0069**	0.0299*	0.0205*

Heat Resistance			
**Strain**	**Time (min)**		
	**10**	**20**	**30**
sspA	0.1105	0.0052**	0.0188*
sspB	0.9998	0.9956	0.7972
sspC	0.9998	0.2097	0.2486
acuC	0.0342*	0.0218*	0.0302*
srtN	0.3385	0.1939	0.2043
ydgE	0.6462	0.9997	0.7802
yfmK	0.5513	0.4652	0.4965
ydgE yfmK	0.6400	0.3540	0.6041

Supplementary Table S3. A table of the corresponding p-values for the data presented in Figures 2 and S1. *B. subtilis* spores were isolated following growth in sporulation media for 48 hours. Spores were diluted and subsequently exposed to heat for a total of 30 minutes. At 10 minute intervals, spores were plated for viable counts, and the survival percentage was calculated as heat-resistant colony forming units (CFU)/ml divided by total viable counts (CFU/ml), pre-heat treatment. Statistical analyses were performed using GraphPad Prism 9. Two-factor ANOVAs with repeated measures, and post-hoc Dunnett square analyses were used to determine statistical significance**.** A p-value of ≤ 0.05 was considered significant. *p<0.05, **p<0.01, ***p<0.001, ****p<0.0001.

1.4 Supplementary Table S4: Formaldehyde resistance p-values.

Formaldehyde Resistance			
Strain	Time (min)		
	10	20	40
hbsK3Q	0.9871	0.0046**	0.0480*
hbsK18Q	0.9998	0.4847	0.2715
hbsK37Q	0.8813	0.5009	0.0110*
hbsK41Q	0.0703	0.0297*	0.0128*
hbsK75Q	0.7420	0.3005	0.0216*
hbsK80Q	0.0648	0.0268*	0.0096**
hbsK86Q	0.3110	0.1011	0.1552

Formaldehyde Resistance			
Strain	Time (min)		
	10	20	40
hbsK3R	0.5181	<0.0001****	0.0170*
hbsK18R	0.4431	0.6517	0.1751
hbsK37R	0.4174	0.0229*	0.0039**
hbsK41R	0.1051	0.2506	0.0822
hbsK75R	0.0817	0.0366*	0.0187*
hbsK80R	0.9728	0.0633	0.0157*
hbsK86R	0.9458	0.5951	0.2836

Formaldehyde Resistance			
Strain	Time (min)		
	10	20	40
sspA	0.0099**	0.0005***	0.0159*
sspB	0.5884	0.1953	0.1400
sspC	0.8789	0.0004***	0.0791
acuC	0.0492*	0.0032**	0.0408*
srtN	0.0192*	0.0011**	0.0114*
ydgE	0.9997	0.0027**	0.2322
yfmK	0.9841	0.0303*	0.0689
ydgE yfmK	0.7916	0.0724	0.2336

Supplementary Table S4. A table of the corresponding p-values for the data presented in Figures 3 and S2. *B. subtilis* spores were isolated following growth in sporulation media for 48 hours. Spores were diluted and subsequently exposed to 2.5% formaldehyde for a total of 40 minutes. At 10, 20 and 40 minutes, spores were plated for viable counts, and the survival percentage was calculated as formaldehyde-resistant colony forming units (CFU)/ml divided by total viable counts (CFU/ml), pre-treatment. Statistical analyses were performed using GraphPad Prism 9. Two-factor ANOVAs with repeated measures, and post-hoc Dunnett square analyses were used to determine statistical significance. A p-value of ≤0.05 was considered significant. *p<0.05, **p<0.01, ***p<0.001, ****p<0.0001.

1.5 Supplementary Table S5: UV resistance p-values.

UV Resistance			
**Strain**	**Time (min)**		
	**1**	**3**	**5**
hbsK3Q	>0.9654	0.9978	0.4688
hbsK18Q	0.4845	0.0202*	0.0652
hbsK37Q	0.9494	0.6172	0.4919
hbsK41Q	0.7120	0.9131	0.9997
hbsK75Q	0.9997	0.9997	0.9996
hbsK80Q	0.5642	0.2376	0.0164*
hbsK86Q	0.0941	0.3481	0.2907

UV Resistance			
**Strain**	**Time (min)**		
	**1**	**3**	**5**
hbsK3R	0.0061**	0.1417	0.0030**
hbsK18R	0.9728	0.1412	0.0310*
hbsK37R	0.7853	0.3256	0.0158*
hbsK41R	0.9716	0.8083	0.6654
hbsK75R	0.8298	0.1095	0.0667
hbsK80R	0.0248*	0.0741	0.0628
hbsK86R	0.0591	0.0054**	0.0034**

UV Resistance			
**Strain**	**Time (min)**		
	**1**	**3**	**5**
sspA	0.1825	0.0138*	0.0004***
sspB	0.6177	0.6415	0.3490
sspC	0.032*	0.2202	0.0842
acuC	0.5749	0.2032	0.1963
srtN	0.6508	0.2452	0.0655
ydgE	0.3446	0.8584	0.9728
yfmK	0.9926	0.1356	0.0422*
ydgE yfmK	0.9100	0.2438	0.0718

Supplementary Table S5. A table of the corresponding p-values for the data presented in Figures 4 and S3. *B. subtilis* spores were isolated following growth in sporulation media for 48 hours. Dilutions of spores were exposed to UV light for 1, 3 and 5 minutes. The percentage of survival was calculated as UV-resistant colony forming units (CFU)/ml divided by total viable counts (CFU/ml), pre-treatment. Statistical analyses were performed using GraphPad Prism 9. Two-factor ANOVAs with repeated measures, and post-hoc Dunnett square analyses were used to determine statistical significance. A p-value of ≤ 0.05 was considered significant. *$p<0.05$, **$p<0.01$, ***$p<0.001$.

2 Supplementary Figures

2.1 **Supplementary Figure S1:** Heat survival curves of spores from mutants that alter the acetylation state of HBsu.

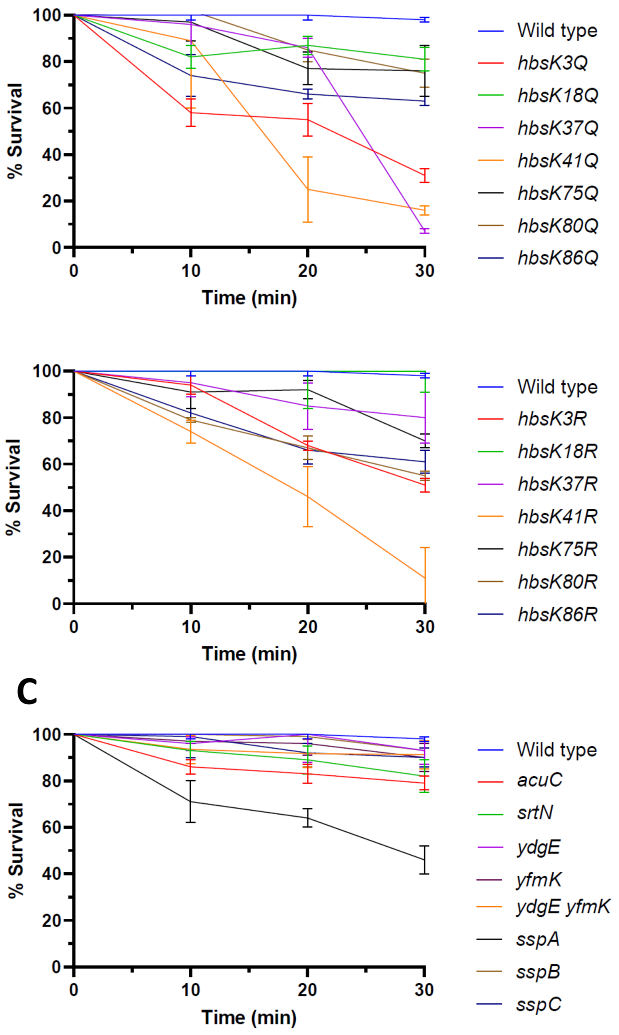

Supplementary Figure S1. Spore suspensions were incubated at 85°C for a total of 30 minutes, with samples taken at 0, 10, 20, and 30 minutes to determine the percentage of survivors. The survival percentage was calculated as heat-resistant colony forming units (CFU)/ml divided by total viable counts (CFU/ml), pre-heat treatment. Displayed are the average percentages determined from three independent replicates, with error bars representing standard deviations. Survival curves of the acetylation mimic mutant spores are displayed in panel (A): Wild type (BD630), *hbsK3Q* (BD8577), *hbsK18Q* (BD8219), *hbsK37Q* (BD8119), *hbsK41Q* (BD8147), *hbsK75Q* (BD8398), *hbsK80Q* (BD8576), *kbsK86Q* (BD7493). The unacetylated mimic mutant spores are displayed in panel (B): *hbsK3R* (BD8387), *hbsK18R* (BD8190), *hbsK37R* (BD8120), *hbsK41R* (BD8148), *hbsK75R* (BD8333), *hbsK80R* (BD7484), and *hbsK86R* (BD7506). The *ssp*, acetyltransferase and deacetylase mutant spores are shown in panel (C): *acuC* (BD6861), *srtN* (BD7375), *ydgE* (BD7199), *yfmK* (BD7203), *ydgE yfmK* (VCB56), *sspA* (VCB4), *sspB* (VCB5), *sspC* (VCB6).

2.2 Supplementary Figure S2: Formaldehyde survival curves of spores from mutants that alter the acetylation state of HBsu.

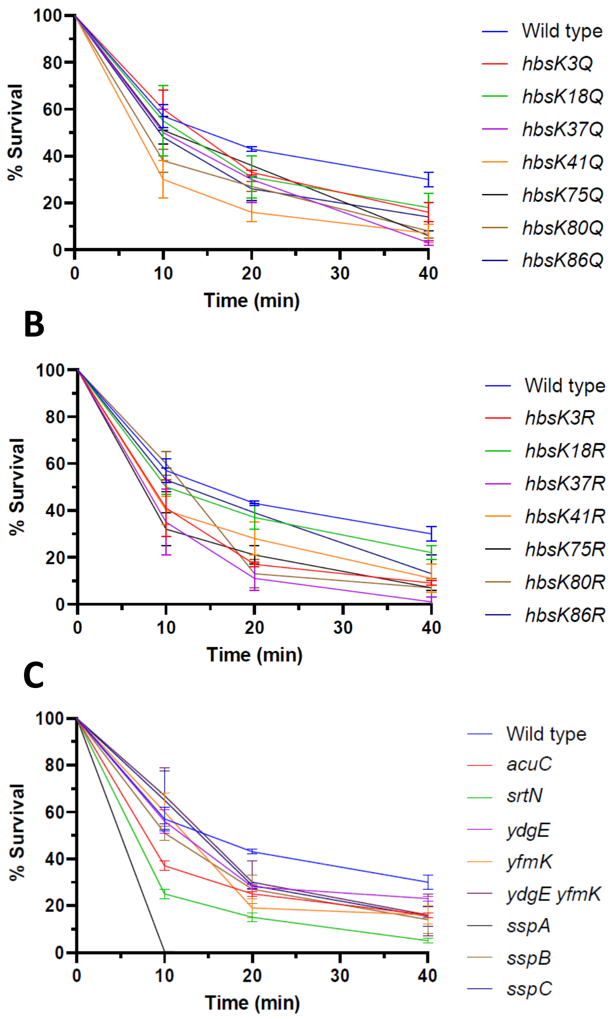

Supplementary Figure S2. Spore suspensions were incubated at 30°C for a total of 40 minutes in the presence of 2.5% formaldehyde. Samples were taken at 0, 10, 20, and 40 minutes and the formaldehyde was neutralized with a 400 mM glycine solution. At each time point, percentage of spores which had survived the treatment were calculated as formaldehyde-resistant colony forming units (CFU)/ml divided by total viable counts (CFU/ml), pre-treatment. Displayed are the average percentages determined from three independent replicates, with error bars representing standard deviations. The strains used in panels (A), (B) and (C) are as described in the legend for Figure S1. For *sspA* spores, there were no survivors after 10 minutes of formaldehyde exposure.

2.3 **Supplementary Figure S3:** UV survival of spores from mutants that alter the acetylation state of HBsu.

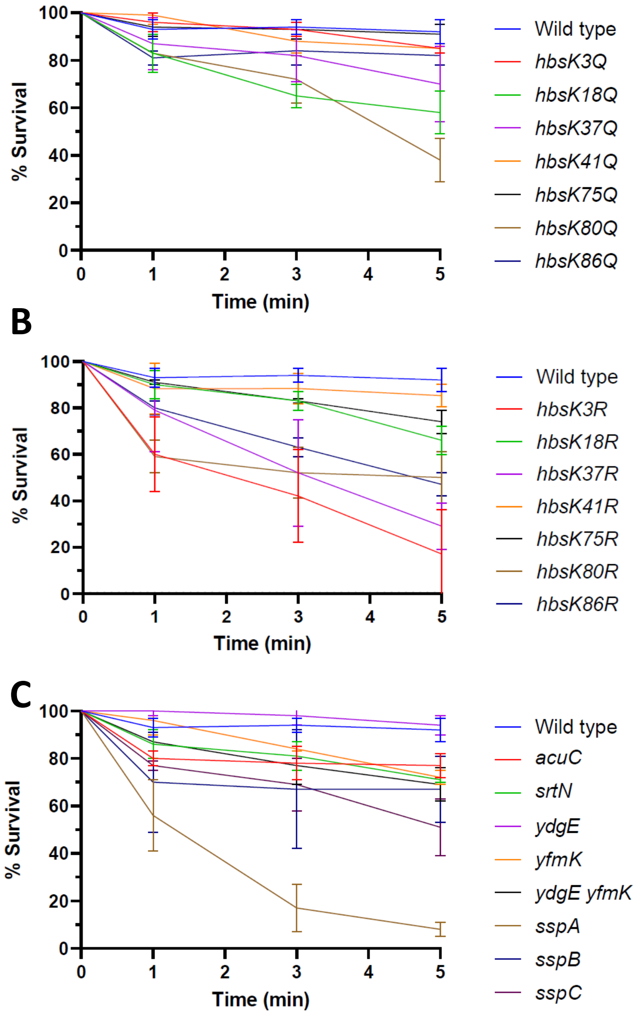

Supplementary Figure S3. Spore suspensions were exposed UV light for 0, 1, 3, or 5 minutes. The percent survival at each time point was calculated as UV-resistant colony forming units (CFU)/ml divided by total viable counts (CFU/ml), pre-treatment. The data displayed is the average percentages determined from at least three independent experiments, with error bars representing the standard deviations. The strains used in panels (A), (B) and (C) are as described in the legend for Figure S1.
